# Genetic Diversity and Spatial Distribution of *Yersinia pestis* by Core Genome-Based Multilocus Sequence Typing Analysis

**DOI:** 10.3390/microorganisms14040898

**Published:** 2026-04-16

**Authors:** Sandra Appelt, Anna-Maria Rohleder, Katarzyna Schmidt, Jacob Gatz, Somayyeh Sedaghatjoo, Holger C. Scholz

**Affiliations:** 1 Centre for Biological Threats and Special Pathogens (ZBS 2), Robert Koch Institute, 13353 Berlin, Germany; 2 Genome Competence Center (MF1), Robert Koch Institute, 13353 Berlin, Germany

**Keywords:** *Yersinia pestis*, plague, multilocus sequence typing, core genome, molecular profiling

## Abstract

*Yersinia pestis* is the etiological agent of plague, a severe and often fatal disease in humans when left untreated. Because of the high genetic clonality of *Y. pestis*, high-resolution genotyping assays are necessary to differentiate between individual strains. Here, we report on the development and validation of a robust and reproducible core-genome multilocus sequence typing (cgMLST) assay for *Y. pestis* comprising 3139 gene targets, enabling high-resolution typing at the strain level. The assay was validated using 222 publicly available *Y. pestis* genomes, including 45 recently sequenced outbreak isolates from Madagascar and 21 isolates from Mongolia. The cgMLST analysis revealed primary clustering aligned with known biovar-associated branches and sub-branches. Additional geographically structured sub-clusters illustrate its application for regional diversification analysis. *Yersinia pestis* strains from different geographic regions were clearly distinguished, consistent with spatial clustering. Within the analyzed dataset, closely related or epidemiologically linked strains differed by zero to three alleles, suggesting this range as an operational reference for identifying highly similar isolates. The cgMLST showed clustering patterns concordant with previously described single-nucleotide polymorphism (SNP) assays. It therefore provides a standardized high-resolution typing approach, with demonstrated applicability for outbreak investigations, source tracking, and comparative genomic surveillance of *Y. pestis*.

## 1. Introduction

*Yersinia pestis* is a Gram-negative rod-shaped bacterium and the etiological agent of plague, a notifiable zoonosis [1]. Plague remains one of the most severe infectious diseases with case fatality rates of 30–100% in untreated patients. Transmission occurs via flea bites, direct contact with infected animals, or inhalation of infectious droplets, resulting in bubonic plague, septicemia and pneumonic plague, respectively. Although largely controlled, in many regions, plague remains endemic in parts of Africa, Asia, and the Americas, where sporadic outbreaks continue to occur [2,3,4]. No autochthonous European cases have been reported since the early 20th century [5].

Genetically, *Y. pestis* is a recently evolved, host-adapted, monomorphic lineage of *Yersinia pseudotuberculosis* [6,7]. Historically, *Y. pestis* is subdivided into the biovars Antiqua, Medievalis, Orientalis, and Pestoides (Microtus) based on differential metabolic traits such as glycerol fermentation and nitrate reduction. However, whole-genome analyses have demonstrated that these phenotypic groupings do not correspond to monophyletic lineages and therefore lack phylogenetic resolution [6,8,9].

During host adaptation, *Y. pestis* underwent genome reduction in its transition from an enteropathogen to a facultative intracellular pathogen. The genome consists of a ~4.65 Mb chromosome and three virulence plasmids (pPCP1, pMT, pCD1), of which pPCP1 and pMT are species-specific [10,11].

*Yersinia pestis* populations are highly clonal and exhibit limited genetic diversity that reduces the applicability of conventional low-resolution typing methods. For example, the classical MLST-7 scheme cannot discriminate between individual *Y. pestis* strains (all *Y. pestis* strains belong to a single Sequence Type, 79) and may not be able to reliably distinguish *Y. pestis* from *Y. pseudotuberculosis* [8,12,13]. Likewise, the available genus-wide Yersinia core-genome multilocus sequence typing assay, based on 500 gene targets [14], also shows insufficient genetic resolution to accurately discriminate between different *Y. pestis* strains. The cgMLST scheme used by enterobase “https://enterobase.warwick.ac.uk/ (accessed on 25 March 2026)” consists of 1553 gene targets. It allows discrimination at the sub-branch level but also lacks sufficient resolution to discriminate between closely related strains within a given lineage. High-resolution typing approaches are therefore required. Whole-genome SNP-based (wgSNP) analyses provide high phylogenetic resolution but are often implemented using study-specific pipelines, limiting inter-laboratory comparability. MLVA achieves high strain discrimination but is limited by inter-laboratory reproducibility and the instability of VNTR loci, which makes an epidemiological interpretation difficult [12,15]. Furthermore, MLVA has limited phylogenetic accuracy at deeper branches and strains with no epidemiological linkage may exhibit identical VNTR profiles. Thus, current approaches either lack sufficient resolution to discriminate closely related *Y. pestis* strains or, despite adequate resolution (e.g., wgSNP, MLVA), lack the standardization, reproducibility, and comparability required for genomic surveillance on a larger scale. A standardized, reproducible genome-wide approach that combines high resolution, robustness, and inter-laboratory reproducibility is therefore needed to ensure accurate genomic characterization of *Y. pestis* for surveillance, biosecurity, risk mitigation, and public health preparedness.

Here, we developed and validated a highly discriminatory cgMLST scheme for *Y. pestis* comprising 3139 core gene targets, providing high resolution while ensuring reproducibility and standardized allele calling. The scheme was applied to 222 publicly available genomes of *Y. pestis* from diverse geographic origins, including isolates from a recent outbreak, to demonstrate its performance and applicability across different datasets, including the assessment of short-term diversification. Cluster analysis was used to define allele-based thresholds for strain discrimination. Performance was evaluated in terms of typeability, discriminatory power, and reproducibility.

## 2. Materials and Methods

### 2.1. cgMLST Target Scheme Definition

The publicly available genome of *Y. pestis* type strain CO92 (NCBI Accession: NC_003143.1) was selected as the seed genome [11]. To determine the cgMLST gene set, a genome-wide gene-by-gene comparison using the cgMLST Target Definer (v.1.4) function of the Ridom SeqSphere+ software (v.7.2.3, Ridom GmbH, Münster, Germany) was performed. The applied default parameters served to exclude certain genes of the seed genome from the cgMLST scheme and to ensure the inclusion of conserved, single-copy, and structurally intact genes suitable for high-resolution typing. The following filters were applied: a minimum-length filter that discards all genes shorter than 50 bp, a start codon filter that discards all genes lacking a valid start codon, a stop codon filter that discards all genes lacking a stop codon or containing multiple stop codons or genes with a stop codon not located at the end of the gene, a homologous gene filter that discards all genes with fragments that occur in multiple copies within a genome (≥90% of identity and >100-bp overlaps), and a gene overlap filter that discards the shorter gene from the cgMLST scheme if the two genes affected overlap by >4 bp.

The remaining genes were included in a pairwise comparison using BLAST (v.2.2.12, parameters: word size 11; mismatch penalty −1; match reward 1; gap open costs 5; gap extension costs 2) against the query genome of selected *Y. pestis* strains. The selected query genomes are publicly available and comprise 10 genomes from *Y. pestis* strains originating from distinct geographical regions and representing diverse molecular profiles, in order to capture the genetic diversity of *Y. pestis*: 1412 (0.PE2, Georgia, NCBI Accession: NZ_CP006783.1); 1522 (0.PE2, Armenia, NCBI Accession: NZ_CP006758.1); Antiqua (1.ANT1, Republic of the Congo, NCBI Accession: NC_008150.1); EV76-CN (1.ORI3, China, NCBI Accession: NZ_CP096666.1); KIM10 (2.MED1, Kurdistan, NCBI Accession: NC_004088.1); MG05-1020 (1.ORI3, Madagascar, NCBI Accession: AAYS01000001.1); 91001 (0.PE4, NCBI Accession: AE017042.1); Nepal516 (2.ANT1, Nepal, NCBI Accession: NC_008149.1); SCPM-O-B-6291 (2.Med, Russia, NCBI Accession: NZ_CP045163.1); and RDC09 (1.ANT1, Republic of the Congo, Short Read Archive (SRA) Accession: SAMN55867701) [6,10,16,17,18,19].

The composition of the resulting cgMLST target set is determined by the selection of seed and query genomes. To minimize potential bias, the query genomes were selected to represent the known phylogenetic diversity of *Y. pestis*, including major branches and biovars.

The final cgMLST scheme was generated by including all genes of the reference genome that were common in all query genomes with a sequence identity of ≥90% and 100% of overlap. Also, all genes that had no start or stop codon in one of the query genomes, as well as genes that had internal stop codons in more than 20% of the query genomes, were discarded. The final cgMLST scheme consisted of 3139 target genes. Information on target genes of the cgMLST assay is provided as Supplementary Table S1 and is made accessible to the public on www.cgmlst.org and as the chewBBACA scheme (https://chewbbaca.online/, accessed on 25 March 2026) [20].

### 2.2. Bacterial Strains of the In-House Collection and Whole Genome Sequencing

Four *Y. pestis* strains (RDC09, Yp15, Yp16, Yp17) of the strain collection at the Robert Koch Institute, Germany, were included in the analyses. These four isolates were sequenced using different sequencing platforms and processed with different assembly strategies, resulting in multiple genome assemblies per strain (20 assemblies in total; see Supplementary Table S2).

For DNA extraction, *Y. pestis* isolates of the in-house collection were grown on Columbia Blood agar plates at 37 °C and harvested after 48 h. The Qiagen Blood and Tissue kit (Hilden, Germany) was used for the extraction of genomic DNA following the supplier’s instructions. All procedures were performed in certified BSL-3 facilities in accordance with institutional and national biosafety regulations.

For samples Yp15, Yp16, and Yp17, libraries were prepared using the Nextera XT DNA Library Preparation Kit (Illumina, San Diego, CA, USA). The resulting libraries were sequenced in paired-end mode (2 × 150 bp) on Illumina MiSeq, NextSeq 550, and NextSeq 2000 platforms (Illumina, San Diego, CA, USA) to evaluate potential differences between sequencing platforms. For RDC09, two libraries were also prepared with the Nextera XT DNA Library Preparation Kit (Illumina, San Diego, CA, USA) and the sequencing in paired-end mode (2 × 150 bp) was performed on MiSeq600 and NextSeq2000 platforms. The sequencing reads obtained for four strains (RDC09, Yp15, Yp16, and Yp17) were de novo assembled with SPADES (version 3.15.5) [21,22] after raw reads quality control trimming, cropping and adapter clipping [23,24]. The assemblies for Yp15, Yp16, and Yp17 were, in addition, polished with pilon (version 1.24) [25]. Sequencing raw data are available in the SRA, Project IDs: PRJNA1426419.

### 2.3. Setting up the Genomic Database

The final genome database consisted of 222 *Y. pestis* genomes, including genomes from the National Center for Biotechnology Information (NCBI) Genome database and the SRA [26,27], and an additional 20 *Y. pestis* genomes from isolates were from the in-house strain collection at the Robert Koch Institute. These genomes were generated on different Illumina sequencing platforms (MiSeq, NextSeq) and assembled using different approaches, with and without polishing.

### 2.4. cgMLST-Based Analysis

To validate the cgMLST scheme and assess its performance, 222 *Y. pestis* genomes (Supplementary Table S2) were analyzed. The genotypic resolution of the assay was evaluated as part of the validation process by analyzing genomes from a panel of *Y. pestis* derivatives differing in biovar lineage and virulence (Antiqua, Angola, CO92, KIM derivatives, EV76 derivatives) obtained from multiple culture collections. A broad geographical representation was achieved by including genomes of *Y. pestis* strains originating from diverse regions, including Armenia, Azerbaijan, China, Georgia, India, Indonesia, Japan, Kazakhstan, Kyrgyzstan, Madagascar, Mongolia, Myanmar, Nepal, Peru, Republic of the Congo, USA, Vietnam and the former USSR (no further localization), reflecting the original metadata provided in the NCBI. In addition, genomes assembled from sequencing data generated using different technologies, including Illumina (HiSeq, MiSeq, NextSeq), Pacific Biosciences (PacBio), 454 Life Sciences, and Sanger sequencing, were included.

For several reference strains, assemblies derived from different sequencing platforms were available, including Angola, Antiqua, CO92, A1122, KIM5 (Angola_1—PacBio/454; Angola_2—Sanger [7]; Angola_LOU—Illumina HiSeq; Antiqua_1—Sanger [10]; Antiqua_2—PacBio/454; CO92_1—Sanger [11]; CO92_2—PacBio; A1122_1—Illumina/PacBio/454; A1122_2—Illumina [28]; and KIM5—Illumina/PacBio/454; KIM5_LOU—454). The corresponding NCBI accession numbers are provided in Supplementary Table S2. To specifically assess the impact of different Illumina sequencing platforms, isolates RDC09, Yp15, Yp16, and Yp17 from the in-house collection were sequenced using the MiSeq and NextSeq2000 platforms and included in the analysis. Also included were genomes previously analyzed using SNP-based phylogenetic analysis [29,30] (Supplementary Table S2) to test for the repeatability of the typing.

The biovar designations in this study were based on previously published SNP-based classifications. For clarity and consistency, strains assigned to the 0.PE4 subgroups such as 0.PE4a and 0.PE4c were grouped under the broader designation 0.PE4 [31]. This approach does not imply strict equivalence between these biovar/phylogroups but reflects an operational grouping applied for comparative cgMLST analysis.

The Pearson chi-squared test with Yates’s correction was applied to test the possibility that identical *Y. pestis* show zero to three allelic differences among each other [32,33]. To enable the use of this test with allelic distance data, pairwise comparisons were converted into a binary variable: strain pairs were assigned a value of 1 if they fell within the defined threshold (0–3 allelic differences) and 0 otherwise. This categorization allowed comparison of the distribution of “within-threshold” versus “outside-threshold” pairs between groups. The Yates’s (1934) correction was applied to avoid overestimation of statistical significance in the small dataset [33]. To run the statistical computing, the free software R (version 4.0.4) was used [34].

The dataset of genomes was divided into two groups. Included in group 1 were strains with identified derivatives (including Kim and EV76) expected to fall within the threshold. It comprised 68 strains, all of which showed zero to three allelic differences within their respective groups. Group 2 comprised unrelated strains without known derivatives to assess the ability of the threshold to distinguish unrelated strains. It comprised 154 strains, of which 9 showed one to three differences from other strains not designated as identical.

### 2.5. Allele-Based Clustering

The allelic profiles were exported from the Ridom SeqSphere+ platform (version 10.0.4) for cgMLST analysis (2, 13) and visualization was performed with GrapeTree (version 1.5.0) [35,36]. The MSTree algorithm [36] was applied with symmetric distance correction, three iterations, and eBURST clustering [37]. Due to differences in handling missing values and the tree-drawing algorithm, the allelic differences between individual strains may slightly differ from the Ridom SeqSphere+ MST results without any impact on the overall tree topology [36,38].

## 3. Results

### 3.1. Overall Assay Performance

The developed cgMLST assay comprises a total of 3139 target genes (63.9% of the total seed genome) that match the stringent parameters described in the Section 2. The accessory genome comprises a further 544 gene targets, which could optionally be used in a hierarchical clustering approach in order to achieve a higher genetic resolution, if necessary. Finally, 404 gene targets did not meet the stringent criteria and were discarded.

Of 222 *Y. pestis* genome sequences, 214 could be genotyped with a high score of “good gene targets” in the range of 93 to 100%. Seven genomes (MNG2883, MNG2197, YN2179, CMCC114001, CMCC71001, C1975003, and YN663) showed a lower but sufficient percentage of good gene targets (90.2–92.8%), still allowing accurate typing with high resolution. One strain, MNG52, only exhibited 73% good targets because of low coverage and was therefore excluded from the analysis.

**Figure 1:**
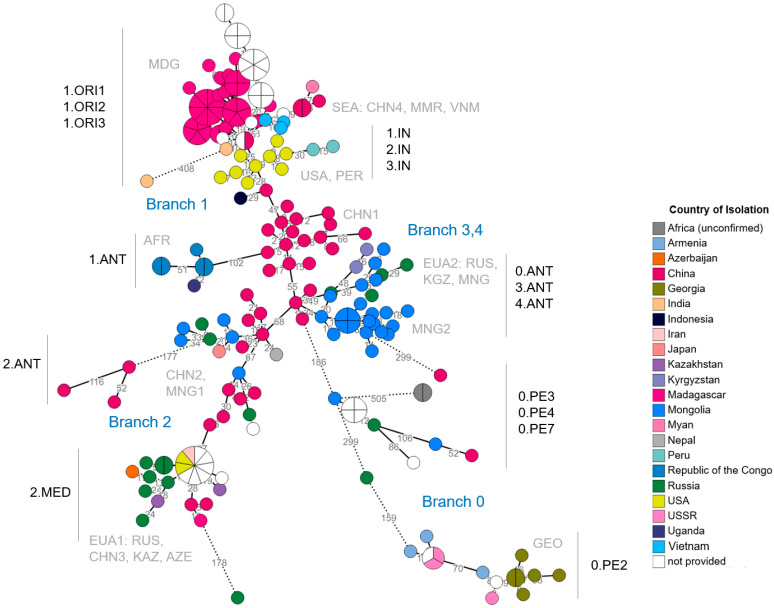
Minimum spanning tree showing the clustering of *Y. pestis* strains based on the cgMLST analysis. Each node represents a distinct cgMLST allelic profile (0–3 allelic differences). The node size refers to the number of genomes with identical allelic profiles. The node color indicates the reported geographical origin. Branches and biovars are shown, and a three-letter code is provided for each identified spatial cluster. Numbers along the branches indicate allelic differences. Branches with more than 150 allelic differences are dotted. Used tool for visualization: GrapeTree. AFR: Africa; AZE: Azerbaijan; CHN: China; EUA: Eurasia; GEO: Georgia; KAZ: Kazakhstan; KGZ: Kyrgyzstan; MDG: Madagascar; MMR: Myanmar; MNG: Mongolia; PER: Peru; RUS: Russia; SEA: Southeast Asia; USA: United States; VNM: Vietnam.

**Figure 2:**
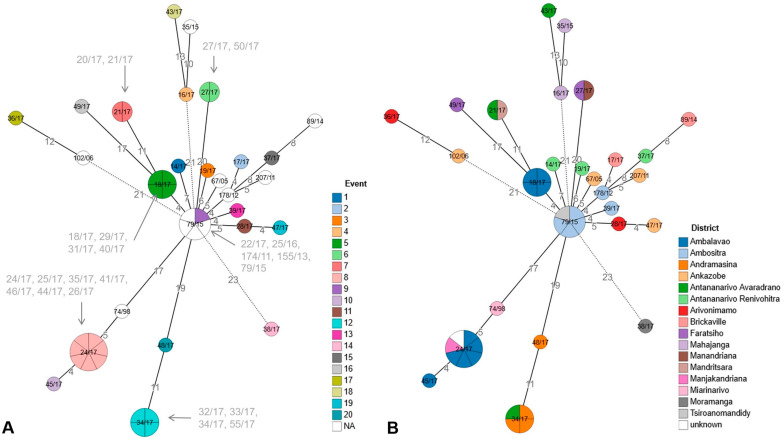
Analysis of outbreak strains, Madagascar, 2017. The minimum spanning tree shows the clustering of *Y. pestis* strains based on cgMLST analysis. Each node represents a distinct cgMLST allelic profile (0–3 allelic differences). Numbers along the branches indicate allelic differences. Branches with more than 20 allelic differences are dotted. Node color indicates the reported event (**A**) or district (**B**). Node size corresponds to the number of genomes sharing identical allelic profiles. For nodes comprising multiple strains, the respective strain IDs are provided [29]. Numbers along the branches indicate allelic differences. Visualization was performed using GrapeTree.

**Figure 3:**
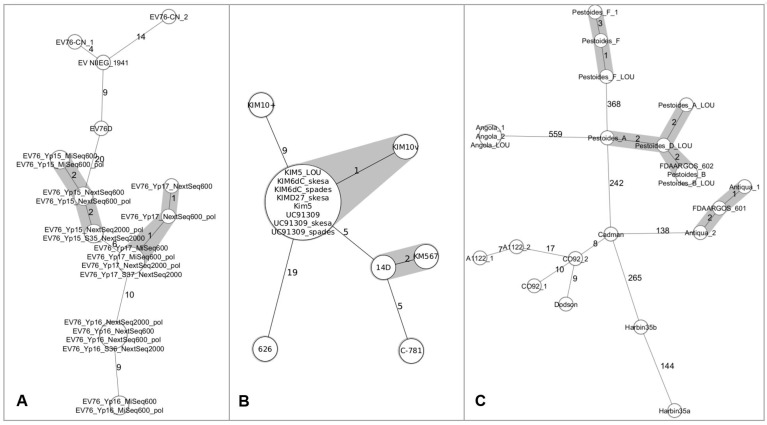
Minimum spanning tree analysis of selected *Y. pestis* strains based on cgMLST allelic profiles. Each node represents a distinct allelic profile; node size is proportional to the number of genomes with identical profiles. The numbers along the branches indicate allelic differences between profiles. Allelic differences of 0–3 are highlighted by grey-shaded connections. (**A**) EV76-related strains, including assemblies generated using different platforms and assembly pipelines. (**B**) KIM-related strains and closely related genomes. (**C**) Comparison of different *Y. pestis* strains, including the Pestoides, Angola, and Antigua clusters. Visualization was performed in Ridom SeqSphere+.

Comparable clustering patterns were observed between the developed cgMLST scheme and previously published SNP-based analyses applied to a subset of 66 *Y. pestis* genomes from Mongolian strains [30] and strains from the Madagascar outbreak in 2017 [29] (Supplementary Table S2). Consistent clustering patterns and comparable genetic resolution were observed between cgMLST-based analyses (this study) and SNP typing [29] (Figure 1 and Figure 2 and Supplementary Table S2). Furthermore, cgMLST profiles of *Y. pestis* strains sequenced using different technologies and assembly strategies (including Angola, Antiqua, KIM5, Pestoides_A, Pestoides_B, Pestoides_F, Yp15, Yp17, RDC09) were fully concordant. These results show that for these assemblies, neither the sequencing platform nor the assembly approach had detectable impacts on cgMLST results. Comparisons between Illumina platforms (MiSeq and NextSeq for, e.g., Yp15, Yp16, Yp17, Figure 3A) showed no systematic impact on allele calling, with the exception of the Yp16 MiSeq assembly, with nine allelic differences from the NextSeq assemblies. These findings show generally consistent allele calling across platforms, but sequencing platforms and assembly strategies may still influence results.

### 3.2. Application of the cgMLST to Population Structure, Lineage Clustering, and Geographic Sub-Structuring

The cgMLST-based distance clustering identified major branches (Branch 0 to Branch 4) that broadly corresponded to classical biovar designations and aligned more closely with lineage structure than with geographic origin (Figure 1). Clustering was primarily observed according to lineages, with additional geographic sub-clustering within lineages [6,39]. Chinese isolates were distributed across both basal clusters and multiple derived lineage-associated branches, including 2.Antiqua (2.ANT) (e.g., 34008, CMCC348002, Harbin35a), 2.Medievalis (2.MED) (e.g., SHAN11, 7338), 1.Orientalis (1.ORI) (e.g., YN663, CMCC114001), 1.Intermedium (1.IN) (e.g., YN472, K21985002), and 0.Pestoides (0.PE) (e.g., Pestoides_F, Pestoides_G) [1,6,39,40,41] (Figure 1 and Supplementary Figure S1).

ANT- and MED-associated lineages were clearly separated from each other by 67 allelic differences [6,39]. In addition, PE-associated lineages (0.PE) formed a deeply divergent cluster that was separated from ANT, IN, and ORI lineages by more than 100 allelic differences. In addition, both 2.MED and 0.PE lineages exhibited limited internal branching, consistent with restricted diversification within these lineages.

Within the Antiqua group, lineage 1.ANT was restricted to Central African isolates (e.g., Antiqua, RDC09, UG05-0454), as previously described. In contrast, lineage 3.ANT occupied a central position within the network and was connected to multiple surrounding lineages, including 2.ANT, 1.IN, and 0.PE. Isolates within 3.ANT displayed pronounced geographic structuring, with African, Mongolian, and Chinese sub-clusters separated by substantial allelic distances. For example, African and Mongolian 3.ANT isolates differed by approximately 55–65 cgMLST alleles, while allelic differences between some Asian and African sub-clusters exceeded 100 alleles.

More recently diverged lineages, including 1.IN and 1.ORI, exhibited lower allelic differences among strains. Lineage 1.IN occupied an internal position, with most strains differing by 6–21 alleles, although one isolate (D106004) differed by 66 allelic differences from other members of the cluster. In contrast, the Orientalis lineages formed a compact cluster characterized by minimal allelic variation, with a maximum of 20 allelic differences between strains. Also, the sub-branches 1ORI1, 1ORI2 and 1ORI3 with several distinct lineages within each sub-branch could be distinguished with the cgMLST assay.

Geographic sub-structuring was identified within several branch-associated lineages (Figure 1, Supplementary Figure S1). Isolates originating from the same geographic regions frequently formed sub-clusters within lineages, for example, Madagascar-associated clusters within 1.ORI, African clusters within 1.ANT, and Mongolian clusters within 3.ANT and 4.ANT, with limited overlap between regions. Distinct regional clustering was evident for isolates from endemic areas, including Madagascar (e.g., MG05_1020, 20/17 and 102/06), China (e.g., YN472 and K21985002), Mongolia (e.g., MNGn29977 and MNGn30012), and the Americas (e.g., Shasta, Cadman8 from the United States and INS_Peru, PY65 from Peru). Within individual lineage-associated branches, isolates from Madagascar formed well-defined sub-clusters that were clearly separated from strains originating from other regions (e.g., Madagascar 1.ORI vs. China 1.ORI, ~15–20 allelic differences; Madagascar 1.ORI vs. the Americas 1.ORI, ~18–22 allelic differences), consistent with local diversification within endemic foci [16,42,43,44,45]. Chinese isolates also grouped into geographically coherent sub-clusters within several lineages, with allelic differences of ~40–60 within 3.ANT-associated lineages and ~10–20 within 1.IN-associated lineages.

Together, these results show the applicability of the cgMLST for characterizing the general population structure of *Y. pestis*, including lineage-associated differentiation and geographic sub-structuring.

### 3.3. Comparative Analysis of Strain Derivatives Across Culture Collections

Within the KIM cluster, KIM5, KIM62C, KIMD27, UC91309 (laboratory-acquired infection), and KIM10V clustered with a maximum of one allelic difference (KIM10V). Strain 14D (Russian origin, flea isolate) was separated by five allelic differences but grouped near KM567 and C-781 (Russian origin, Citellus musticus), within the KIM-associated cluster. KIM10+ was positioned nine allelic differences away from the central KIM node, while strain 626 (human isolate, 1945) was more distantly related (19 allelic differences), yet remained within the broader KIM cluster (Figure 3). This observation is in line with previous descriptions of KIM-derived laboratory strains [46,47].

Pestoides formed two well-separated clusters. One cluster comprised Pestoides_A, _B, and _D strains with 1–3 allelic differences, while the second cluster was well distinct by 368 alleles and consisted exclusively of Pestoides_F strains.

Three Antiqua genomes from different repositories were identical (0 alleles). FDAARGOS_602 was identical to Pestoides_B despite lacking annotation, and FDAARGOS_601 clustered with Antiqua without being designated accordingly. Angola_1, Angola_2, and Angola_LOU from different repositories were likewise identical (0 alleles) (Figure 3C).

CO92 genome assemblies (CO92_1: GCF_000009065.1 and CO92_2: GCF_001293415.1) differed by 10 alleles. The PacBio-only assembly showed no increased completeness compared with the Sanger assembly (three vs. four contigs; 3139 good cgMLST targets); both assemblies clustered within the CO92 group. Cadman (eight allelic differences) clustered within this group and is a known CO92 derivative, whereas Dodson (nine allelic differences) also clustered nearby but has not been formally described as such. The Dodson strain clustered in close proximity to CO92 and was isolated in Arizona, a neighboring state to Colorado.

Two generated A1122 genome assemblies (2011 vs. 2015), derived from Illumina-only and hybrid sequencing data, differed by seven alleles. Similarly, two assemblies of EV76-CN (EV76-CN_1: GCF_024758685.1 and EV76-CN_2: GCF_000324805.2; Supplementary Figure S1A), deposited as identical strains, differed by more than 14 alleles associated with differences in sequencing coverage (432× vs. 59×).

Harbin35 (Harbin35a: GCF_000186725.1; deposited 2011, with four contigs, 16× average coverage) and Harbin 35 (Harbin35b: GCF_000834275.1; deposited 2014, with four contigs, 366× average coverage) differed by approximately 144 allelic differences (Figure 3C) associated with different sequencing coverages.

Based on the observed clustering patterns, allelic differences of one to three alleles were compatible with a single strain or closely related strain derivatives within the context of the dataset analyzed in this study. The suitability of this cutoff, defined as allelic differences of zero to three alleles between single-strain isolates and closely related derivatives, was statistically evaluated using Pearson’s chi-square test with Yates’s continuity correction. The chi-square showed a statistically significant difference between the two groups (X^2^ 67.617; *p* = 2.12^−16^), indicating that allele differences from zero to three alleles were associated with confirmed strain derivatives.

### 3.4. Application of the cgMLST to Outbreak Strain Investigation and Genetic Variability

The cgMLST-based analysis of outbreak-associated *Y. pestis* isolates from the Madagascar 2017 outbreak showed a high degree of allelic similarity among epidemiologically linked strains (Figure 2A). Genomes were grouped in accordance with the different described emergences, events 1 to 20. In this context, the isolates 18/17, 29/17, 31/17, and 40/17, which were assigned to event 5 and sampled between mid-September and late October 2017, clustered in a single node in the cgMLST analysis and showed no allelic differences, consistent with a single outbreak-related transmission cluster from Tsinjoarivo Imanga, as described by Andrianaivoarimanana, V. et al., 2024 [29]. This outbreak cluster was separated by four allelic differences from strain 22/17. Other isolates located at the same central node, with isolate 22/17 (e.g., 25/16, 174/11, 155/13, 79/15), originated from earlier years and were distinct from the main outbreak cluster. In addition, Figure 2B provides a spatial context for the outbreak isolates by indicating their districts and communes of origin, illustrating the potential of cgMLST-based analyses to support the reconstruction of transmission routes when combined with epidemiological information. For example, isolate 22/17 (event 9) originated from Manjakandriana/Ranovao; the patient was transferred to Antananarivo for treatment, and our analysis geographically localizes this isolate outside the capital.

A further example is provided by event 8, where the isolates 24/17, 25/17, 35/17, 41/17, 46/17, 44/17, 26/17 form a single cluster with no allelic differences. Isolate 35/17 from this cluster was obtained from a Madagascar tourist. Despite lacking travel details, previous findings suggested that exposure may have occurred in Tsiroanomandidy, which clustered in the same cgMLST node. In contrast, the isolate associated with event 1 (14/17) from Miarinarivo differed by more than seven allelic differences from two other isolates from the same region (19/17 and 37/17), which were associated with events 3 and 15. These findings indicate that multiple *Y. pestis* strains were circulating within the same geographic area during the outbreak.

## 4. Discussion

*Yersinia pestis* is a monomorphic clone that diverged recently from *Y. pseudotuberculosis* with limited genetic diversity, underscoring the need for high-resolution typing approaches for microbial profiling, strain identification, and epidemiological investigations. Genome-wide SNP analyses previously demonstrated that, despite its clonality, *Y. pestis* shows a well-defined hierarchical population structure with distinct radiations and geographically structured lineages [6,30,39,48,49]. These analyses enabled high-resolution strain identification but are not standardized across laboratories. With the cgMLST scheme developed here, the known population structure of *Y. pestis* could be reproduced [6,8]. Clustering into the main branches (0–4) and sub-branches up to the specific phylogenetic lineages broadly aligned with SNP-based phylogenetic structure. Allele-based profiling showed patterns similar to SNP-based analysis, with cgMLST clustering aligning with the overall SNP-based phylogenetic structure while providing a standardized and comparable framework for inter-laboratory analysis.

Although the composition of the cgMLST scheme is determined by the selected seed and query genomes used for its definition, the consistent performance observed across the analyzed dataset indicates consistent performance within this dataset and does not allow conclusions regarding the impact of genome selection on other datasets.

The basal positioning of Pestoides (0.PE) lineages in our dataset, with their clear separation from ANT, MED, IN, and ORI lineages by >100 allelic differences, aligns with previous descriptions of divergence models [6,8,39]. ANT- and MED-associated lineages were clearly separated from each other by 67 allelic differences, in agreement with the divergence previously reported in genome-wide analyses [6,39]. The compact clustering of 1.ORI and 1.IN in our dataset indicates limited diversification within these lineages, reflecting recent expansions during the Third Pandemic radiation [6,16,39,42,45,50]. These findings indicate that cgMLST captures both deep ancestral divergence and recent evolutionary patterns while providing a standardized and portable framework for comparative analysis.

Lineage- and biovar-associated clustering represented the primary level of population structure, whereas geographic sub-structuring was observed as a secondary pattern within established lineages. Although clear regional clustering was evident, particularly within endemic foci, the population structure was more strongly associated with lineage affiliation than with geographic origin. This pattern is consistent with ecological models of plague persistence in geographically isolated natural foci [4,30,42,50,51]. The region-specific clustering observed in Madagascar, Mongolia, China, and the Americas reflects previously described country- or focus-specific radiations [6,8]. In particular, Madagascar genomes represent a well-documented radiation derived from the Third Pandemic lineage with a compact sub-clustering of strains in our cgMLST analysis supporting the historical expansion model [6,16,42,43]. Also, the broad distribution of Chinese isolates across both basal and derived branches points to the role of China, and more broadly East Asia, as a reservoir of *Y. pestis* diversification and a potential evolutionary reservoir [1,6,39,40,41].

This observation is consistent with phylogenetic reconstructions suggesting that *Y. pestis* evolved in or near China, followed by global radiations [6,39,49]. However, the comparatively high number of Chinese isolates included in this dataset may partially influence this pattern and should be considered when interpreting the geographic structure.

The Madagascar 2017 outbreak strain [29] analysis further shows that the cgMLST can be applied to identify transmission clusters at the outbreak scale. Indistinguishable cgMLST profiles within epidemiologically defined events are in line with previous SNP-based outbreak investigations [6,30,39,49]. Multiple distinct genotypes were observed within the same region during the outbreak, indicating the co-circulation of distinct strains, as described for long-term focus persistence in endemic foci [16,43,49].

Reference strain derivatives stored in different laboratories may accumulate measurable genomic divergence during repeated passage [52,53]. In this context, the KIM-associated genomes analyzed here represent closely related derivatives rather than genetically identical bacterial strains.

Our observation of zero to three allelic differences in the dataset provides an operational reference range for strain delineation using the cgMLST assay developed herein. However, this range should not be considered universally applicable, as it may be influenced by dataset composition, publicly available genomes, sequencing technology, and assembly strategy.

These methodological and data-related factors may also influence the observed allele range and should be considered when interpreting allele-based distances and threshold-based classifications. The large allelic distance observed between two Harbin35 assemblies was associated with differences in sequencing coverage rather than true strain divergence and illustrates the importance of data quality in comparative analysis.

In summary, given the cgMLST reproducibility across sequencing platforms and assembly strategies, it represents a standardized and portable approach for surveillance, outbreak investigation, strain verification, and long-term evolutionary studies of *Y. pestis*. Given its historical weaponization and continued classification as a potential biological threat agent, accurate genomic characterization is also critical for biosecurity aspects and public health preparedness.

## 5. Conclusions

The cgMLST scheme developed here provides a standardized and high-resolution approach for the genomic characterization of *Y. pestis*. It showed consistent results and high reproducibility across sequencing platforms, with clustering patterns broadly consistent with SNP-based analysis.

The assay can resolve the population structure and geographic sub-clustering, while an allele range of one to three differences was observed among closely related strains in this dataset and may serve as an operational reference for identifying highly similar isolates and potential outbreak clusters. However, this range is dataset-dependent and should not be interpreted as a universally applicable threshold.

Overall, this cgMLST scheme represents a standardized and portable tool, with demonstrated applicability for the surveillance, outbreak analysis, and strain verification of *Y. pestis*.

## Data Availability

Sequencing data generated during this work are available at Short Read Archive, Project ID: PRJNA1426419. The cgMLST is made accessible to the public on www.cgmlst.org and is available as a chewBBACA schema.

## Supplementary Materials

The following supporting information can be downloaded at: https://www.mdpi.com/article/10.3390/microorganisms14040898/s1: Table S1. The cgMLST loci. Table S2. Information on *Y. pestis* genomes included in the analysis. All listed genomes are available in public databases. Figure S1. Minimum spanning tree analysis of *Y. pestis* strains based on cgMLST allelic profiles. Each node represents a distinct allelic profile; node size is proportional to the number of genomes sharing an identical profile. Numbers along the branches indicate allelic differences between profiles. (A) Nodes labeled with strain IDs. (B) Nodes colored according to biovar classification. Visualization was performed using GrapeTree.

## Abbreviations

The following abbreviations are used in this manuscript:

SRA	Short Read Archive
SNP	Single Nucleotide Polymorphism
cgMLST	core genome Multilocus Sequence Typing
NCBI	National Center for Biotechnology Information
wgSNP	whole-genome Single Nucleotide Polymorphism
