# Supplementary figures and images for: Genetic Diversity and Spatial Distribution of Yersinia pestis by Core Genome-Based Multilocus Sequence Typing Analysis
Source: Microorganisms. 2026 Apr 16;14(4):898. doi: 10.3390/microorganisms14040898 (PMC13118440; doi:10.3390/microorganisms14040898)

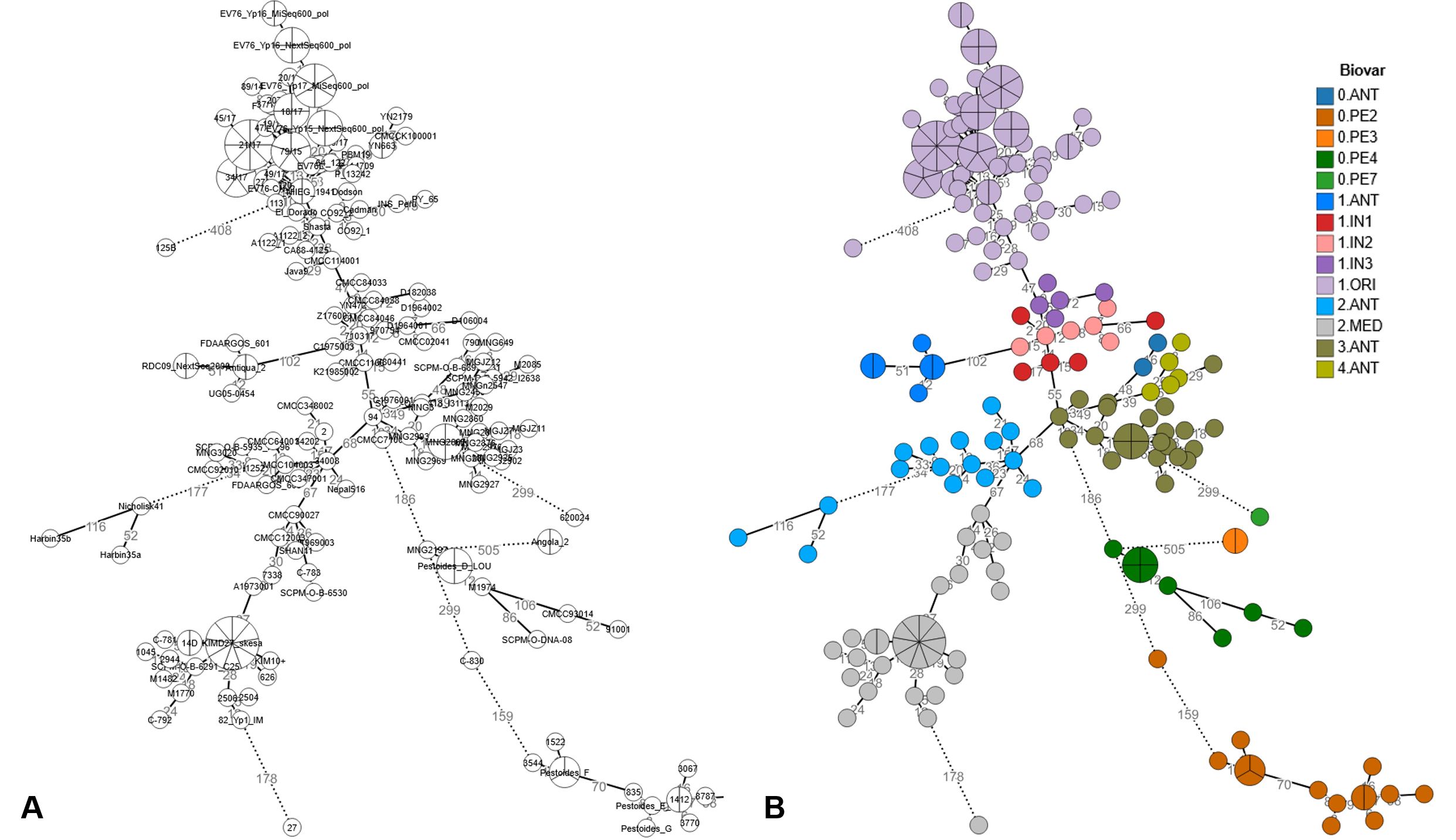

Supplement: Supplementary file 1 [file microorganisms-14-00898-s001.zip › Supplementary_Figure_S1.JPG]
